# La syphilis congénitale révélée par une fracture spontanée

**Published:** 2011-11-22

**Authors:** Mounia Lakhdar Idrissi, Leila Ismaili, Abdelhak Bouharrou, Moustapha Hida

**Affiliations:** 1 Service de Pédiatrie, CHU Hassan II de Fès, Maroc; 2 Service de Néonatologie et de Réanimation Néonatale, CHU Hassan II de Fès, Maroc

**Keywords:** Syphilis, congénitale, fracture, sérologie

## Abstract

Alors qu'elle est actuellement oubliée dans les pays développés, la syphilis congénitale se voit encore chez nous faute du dépistage anténatal. Ses formes cliniques sont polymorphes et orientent à tord vers d'autres pathologies surtout en période néonatale. Le diagnostic n'est donc pas toujours facile. La révélation d'une syphilis congénitale par une fracture spontanée est exceptionnellement décrite. Nous rapportons dans ce travail le cas d'un nourrisson de 2 mois ramené en consultation pour limitation douloureuse des mouvements du bras droit. Le diagnostic est évoqué sur les données radiologiques et confirmé par la sérologie syphilitique. Le traitement a reposé essentiellement sur l'administration de la pénicilline G avec une bonne évolution clinique.

## Introduction

Les manifestations cliniques de la syphilis congénitale sont multi systémiques et non spécifiques. L'atteinte squelettique type ostéochondrite est fréquente surtout chez les nourrissons symptomatiques, elle se constitue en fin de grossesse ou lors des trois premiers mois de la vie et siège le plus souvent au niveau du coude de façon bilatérale et symétrique. Au début elle se manifeste par une douleur à la mobilisation puis apparaît le stade de la pseudo-paralysie avec une impotence douloureuse.

## Observation

Nous rapportons le cas d'un nourrisson de 2 mois et 22 jours, adopté à l’âge d'un mois, sa mère biologique est célibataire et nous n'avons pas d'idée sur le déroulement de la grossesse ni de l'accouchement. Le nourrisson a été ramené en consultation pour une pseudo-paralysie du membre supérieur droit ayant été constatée dès le début mais qui s'est progressivement accentuée. Il n'y avait pas de notion de traumatisme ni de notion de maltraitance de la part de la famille adoptive. L'examen clinique à son admission a trouvé: un poids à 3650 grammes (-1DS), une taille à 51cm (normale) et un périmètre crânien à 37cm (normal), une température à 36,6°C, une saturation en oxygène à 98% à l'air ambiant et un dextro à 0,8g/l. Le nourrisson avait un teint grisâtre et discrètement pale mais il était tonique, réactif et stable sur le plan hémodynamique et respiratoire. Son membre supérieur droit était tombant et douloureux à la palpation, la mobilité de la main du même côté était conservée mais faible. Les autres membres étaient libres; pas d'autres douleurs provoquées à la palpation articulaire ou osseuse. Par ailleurs, son examen neurologique était normal avec des réflexes archaïques présents et une fontanelle antérieure normo-tendue. L'examen abdominal n'avait pas objectivé d'hépato ni de splénomégalie. Son examen cutané ne montrait pas d’éruption particulière et le reste de son examen somatique était sans particularité.

**Figure 1:**
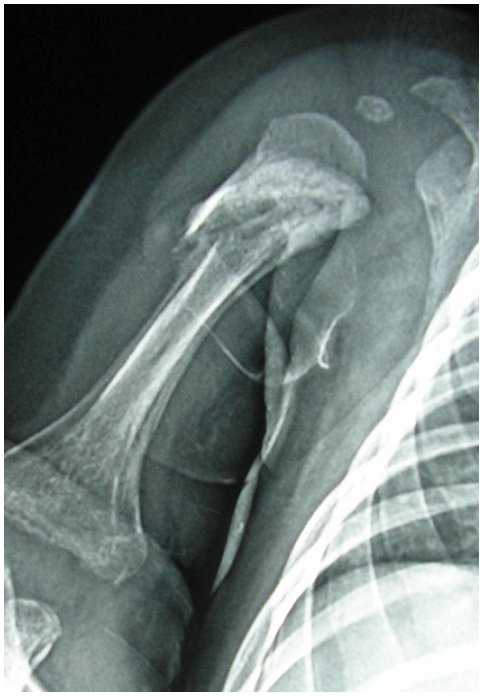
Radiographie montrant une fracture de l'humérus droit chez un 2 mois et 22 jours pris en charge pour syphilis congénitale révélée par une fracture spontanée

**Figure 2:**
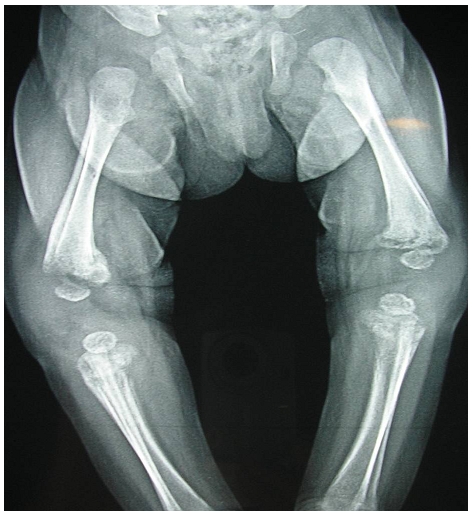
Radiographie montrant une ostéolyse des genoux et une périostite chez un 2 mois et 22 jours pris en charge pour syphilis congénitale révélée par une fracture spontanée

La radiographie du membre supérieur droit avait trouvé une fracture au niveau du tiers supérieur de l'humérus droit, une ostéolyse diffuse des deux extrémités humérales avec une réaction périostée bilatérale importante (Figure 1). Le bilan radiologique a été complété par une radiographie du squelette qui a montré le même aspect d'ostéolyse et de périostite au niveau des deux fémurs (Figure 2). La fracture, semblant alors être pathologique sur une ostéochondrite, a fait suspecter une syphilis congénitale en première intention.

La possibilité d'une ostéogénèse imparfaite était écartée devant la présence de l'ostéolyse et le caractère unique de la fracture. En effet, la sérologie syphilitique IgM réalisée chez lui était positive avec un VDRL supérieur à 1/60 et un TPHA supérieure à 2560. Le statut maternel était méconnu. A la numération formule sanguine on a noté un taux de globules blancs à 19 000/mm^3^ avec un taux de polynucléaires neutrophiles à 9170 et un taux de lymphocyte à 6100; une anémie à 8.4 g /dl normochrome normocytaire et un taux de plaquette correct. La CRP était à 50 mg/l. La ponction lombaire était normale. La sérologie VIH était non négative. Le bilan hépatique était sans anomalie. Sur le plan thérapeutique le nourrisson a été mis sous pénicilline G à raison de 100 000UI /kg/j pendant 10 jours avec un bandage orthopédique pour son bras droit. L’évolution était favorable avec une amélioration du teint, une récupération progressive et lente de la mobilité du membre supérieur droit et une normalisation de la CRP à la fin du traitement. L'amélioration radiologique n'est pas encore constatée.

## Discussion

La syphilis congénitale (SC) est devenue actuellement une pathologie très rare dans les pays développés. Elle demeure une forme préoccupante de la syphilis [1]. Les facteurs responsables de sa persistance sont la recrudescence des maladies sexuellement transmissibles et l'absence ou l'insuffisance des suivis d'un grand nombre de grossesses dans notre pays [2,3]. En effet, seulement 17% des femmes enceintes font une vérification sérologique en début de grossesse. De même la mauvaise observance thérapeutique maternelle augmente le risque de transmission mère-enfant. Ce risque est d'autant plus important chez les nouveau-nés prématurés ou hypotrophes [4,5].

Les tableaux cliniques sont polymorphes, allant d'un tableau complet à une atteinte pauci-symptomatique. Ils peuvent comporter des lésions cutanéo-muqueuses ou multi-viscérales notamment hépatique et hématologique ou des lésions ostéoarticulaires [6]. Ces dernières se présentent généralement sous forme d'ostéochondrite qui est présente dans 80% des cas. Cependant, la révélation d'une SC par une fracture osseuse est exceptionnellement décrite dans la littérature. La survenue de l'atteinte ostéoarticulaire est le plus souvent précoce de la naissance au 3ème mois de la vie. Les lésions sont bilatérales et symétriques avec une atteinte surtout des membres supérieurs. Elle se manifeste sous deux formes: la forme latente et la forme inflammatoire douloureuse qui est responsable d'une pseudoparalysie dite de Parrot [7].

La radiographie standard met en évidence l'atteinte des métaphyses des os longs. Les lésions passent par quatre stades où le stade I comporte un épaississement métaphysoépiphysaire, le stade II comprend des bandes claires au même endroit, le stade III est caractérisé par le signe de Wimberger qui est une encoche du bord interne du tibia et le stade IV qui est exceptionnel comporte une fracture métaphysaire comme le cas de notre patient. La périostite est latente, elle peut prendre trois aspects: périostite calleuse, en bulbe d'oignon ou engainante L'ostéomyélite est également rare et latente.

D'autres formes cliniques sont encore plus rares. La SC peut être révélée en anténatale par une anomalie du rythme cardiaque fœtal à type de bradycardie prolongée témoignant généralement d'une chorioamniotite syphilitique sévère [8]. Dimitriou a rapporté un cas de SC diagnostiqué sur une détresse respiratoire d’évolution atypique et d’étiologie indéterminée avec une radiographie thoracique montrant des infiltrats bilatéraux diffus et une sérologie positive [9].

Sur le plan biologique, la combinaison du VDRL (détection d′anticorps anti-cardiolipides, hétérospécifiques) et du TPHA (hémagglutination passive d′antigènes tréponémiques) permet le dépistage avec une sensibilité de 84%. La pratique d′un test d′immunofluorescence spécifique (FTA abs) permet de confirmer précocement le diagnostic. La primo-infection, la réactivation d′une infection latente et la réinfection s′accompagnent de la production d′IgM spécifiques dont la détection est majeure pour le diagnostic de syphilis congénitale [10].

Le traitement de la syphilis congénitale est basé sur la pénicilline G à dose de 100.000 à 150.000 U / kg / j soit 2 à 3 perfusions par jour de 50.000 U / kg pendant une durée de 10 à 14 jours. Ce traitement peut être associé à une corticothérapie pour prévenir une réaction d'Herxheimer due à la lyse brutale des tréponèmes. La Benzathine Pénicilline à la dose de 50.000 U / kg en une injection IM, est réservée aux nouveaux nés à risque de SC mais qui sont asymptomatiques.

## Conclusion

Les lésions ostéoarticulaires sont retrouvées dans 80% des cas de syphilis congénitale. Cependant la survenue de fractures avec une pseudo-paralysie de Parrot reste une forme très rarement observée. La détection et le traitement appropriés à base de la pénicilline et en temps opportun est une intervention très efficace pour réduire la transmission mère-enfant. Plus de recherche sont nécessaires pour identifier les stratégies les plus rentables pour atteindre une couverture maximale de dépistage pour toutes les femmes enceintes et, si nécessaire, faciliter l'accès au traitement.
